# Uncovering a Novel Homozygous MSH6 Variant in a Child Presenting With Glioblastoma: A Case of Constitutional Mismatch Repair Deficiency

**DOI:** 10.7759/cureus.92789

**Published:** 2025-09-20

**Authors:** Aisha Althomali, Nahla Mobarak, Azhar Alshoumer, Tahani Alanazi

**Affiliations:** 1 Anatomic Pathology, King Abdulaziz Medical City, Riyadh, SAU; 2 Pediatric Hematology and Oncology, King Fahad Medical City, Riyadh, SAU; 3 Anatomic Pathology, King Fahad Medical City, Riyadh, SAU

**Keywords:** constitutional mismatch repair, msh6, pediatric high-grade glioma, t-lymphoblastic lymphoma, variant of uncertain significance

## Abstract

Constitutional mismatch repair deficiency (CMMRD) is a rare autosomal recessive cancer predisposition syndrome caused by biallelic mutations in mismatch repair (MMR) genes. We report a previously healthy seven-year-old boy who presented with a high-grade glioma and was later diagnosed with T-lymphoblastic lymphoma (TLL). Immunohistochemistry (IHC) of the brain tumor showed partial loss of MSH6 expression in both tumor and adjacent normal cells. Genetic testing identified a homozygous MSH6 variant (c.3680T>G, p.Ile1227Arg), with both parents confirmed as heterozygous carriers. This variant has not previously been reported in the homozygous state with clear clinical correlation. The combined clinical, histologic, and genetic findings strongly support a diagnosis of CMMRD and fulfill multiple American College of Medical Genetics and Genomics (ACMG) criteria, supporting reclassification of this variant as likely pathogenic. This case is notable for providing one of the few documented homozygous MSH6 mutations with a well-defined childhood CMMRD phenotype. It underscores the importance of integrating clinical, radiologic, histologic, and genetic evidence for accurate diagnosis, guiding surveillance, treatment, and family counseling.

## Introduction

Constitutional mismatch repair deficiency (CMMRD) is a rare autosomal recessive cancer predisposition syndrome caused by biallelic pathogenic variants in mismatch repair (MMR) genes (MSH2, MSH6, MLH1, or PMS2), with MSH6 variants accounting for ~20% of cases [1,2]. In contrast, Lynch syndrome arises from monoallelic (heterozygous) variants in the same genes and typically manifests in adulthood with a narrower tumor spectrum [1].

Children with CMMRD frequently present early with a broad tumor spectrum that includes high-grade gliomas, hematologic malignancies, and gastrointestinal cancers. Nearly half develop brain tumors, with a median age at diagnosis of 9-10 years [3-5].

Phenotypic features such as consanguinity, café-au-lait macules, and axillary freckling can overlap with neurofibromatosis type 1, contributing to diagnostic delays. Given its estimated incidence of ~1 per million births and clinical overlap with more common disorders, recognition requires a high index of suspicion [2,6].

Among CMMRD-associated malignancies, T-lymphoblastic lymphoma (TLL) is noteworthy. This aggressive neoplasm of immature T-cell precursors typically presents in children with mediastinal masses and respiratory symptoms. Although usually sporadic, TLL occurs with increased frequency in CMMRD, with reports suggesting that >10% of pediatric TLL cases may harbor underlying CMMRD, highlighting the distinct biology of pediatric T-cell lymphoid neoplasms [7,8].

From a diagnostic standpoint, microsatellite instability (MSI) testing and MMR immunohistochemistry (IHC) remain essential tools. Nevertheless, MSI may be absent in CMMRD-related brain tumors, and IHC can reveal loss or partial loss of MMR proteins not only in tumor tissue but also in adjacent non-neoplastic cells [1,5].

Here, we describe the case of a child with glioblastoma carrying a previously unreported homozygous MSH6 variant of uncertain significance (VUS). The subsequent development of TLL further supports a diagnosis of CMMRD. Through detailed clinical, radiologic, histologic, and genetic characterization, this case highlights the importance of comprehensive evaluation in suspected CMMRD.

## Case presentation

**Figure 1:**
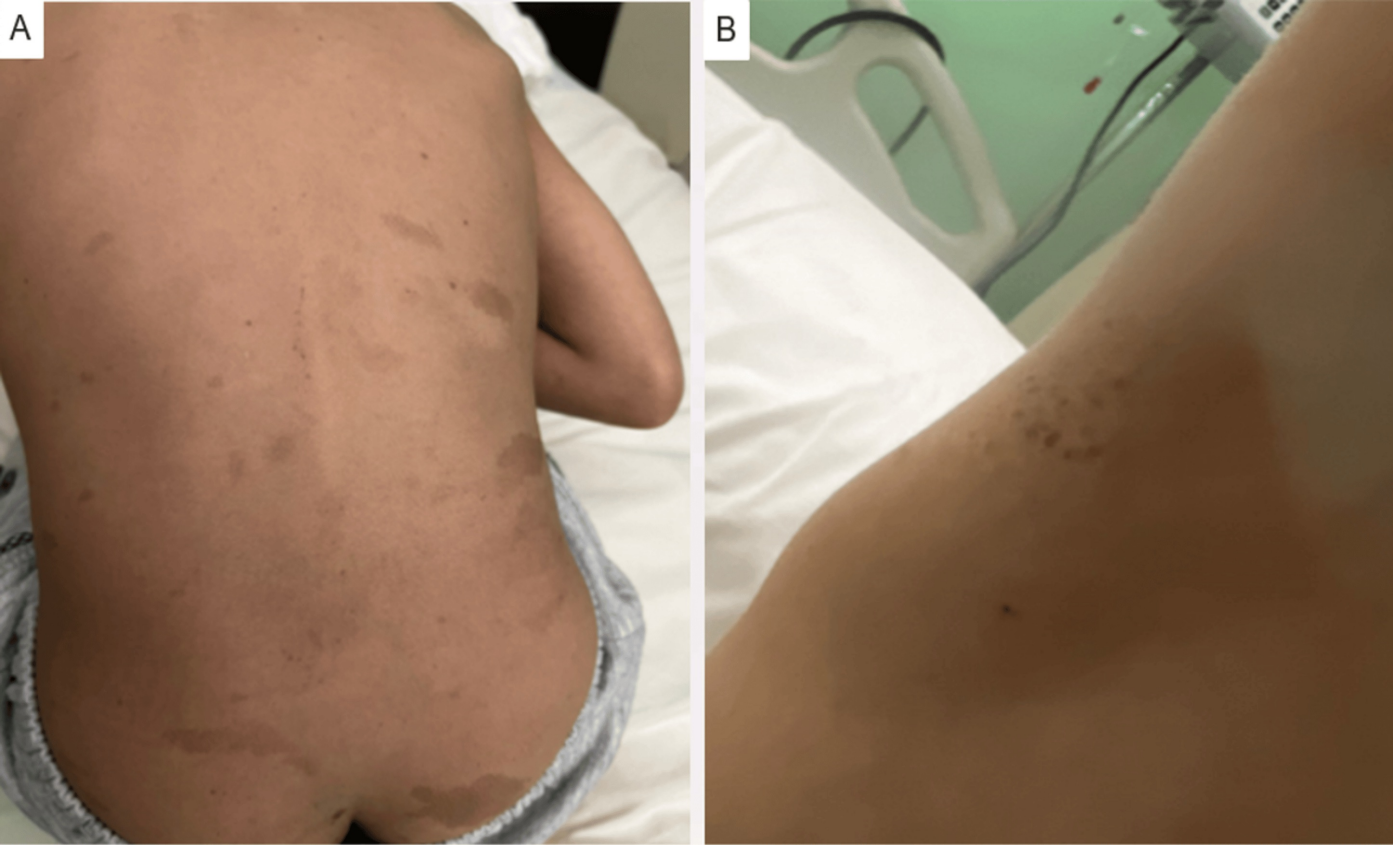
Café-au-lait macules and axillary freckling (A) Multiple scattered café-au-lait macules on the trunk, back, and buttocks. (B) Freckling observed in the right axilla.

A previously healthy seven-year-old boy presented with a three-week history of progressively worsening morning headaches localized to the frontal region, associated with vomiting and generalized fatigue. On physical examination, multiple café-au-lait macules were noted over the trunk, back, and buttocks, along with axillary freckling (Figure 1). 

**Figure 2:**
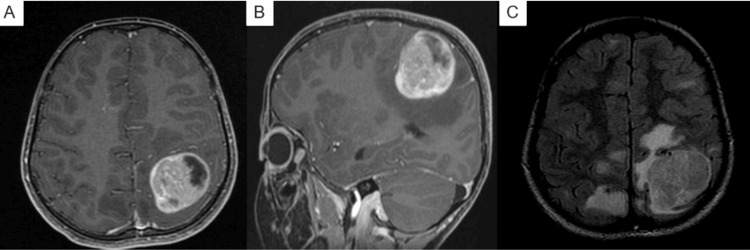
Radiologic findings on brain MRI (A, B) T1-weighted MRI with contrast shows a large ill-defined mixed cystic and solid lesion (4.3 × 3.5 × 3.5 cm) at the left parietal lobe, associated with surrounding white matter swelling. (C) T2-FLAIR image demonstrates multiple bilateral asymmetric subcortical white matter hyperintensities, the largest located at the posterior right parietal lobe.

Given these clinical findings, brain MRI revealed a left parietal lesion with surrounding edema, suspicious for a high-grade glioma (Figure 2).

The patient underwent craniotomy, cortical mapping, and gross total resection of the lesion under general anesthesia.

**Figure 3:**
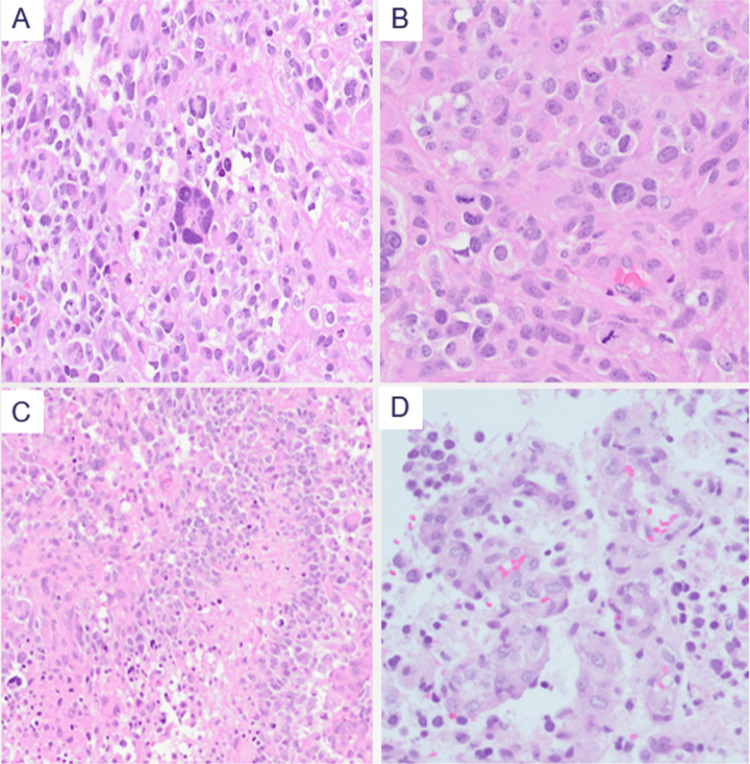
Histopathological features of the brain tumor (H&E stain) (A) Sever nuclear pleomorphism and bizarre multinucleated giant cells. (B) Abundant mitotic figures. (C) Pseudopalisading necrosis. (D) Microvascular proliferation.

Histopathological evaluation revealed features consistent with a high-grade glioma, including severe nuclear pleomorphism, frequent mitoses, pseudopalisading necrosis, and microvascular proliferation (Figure 3).

**Figure 4:**
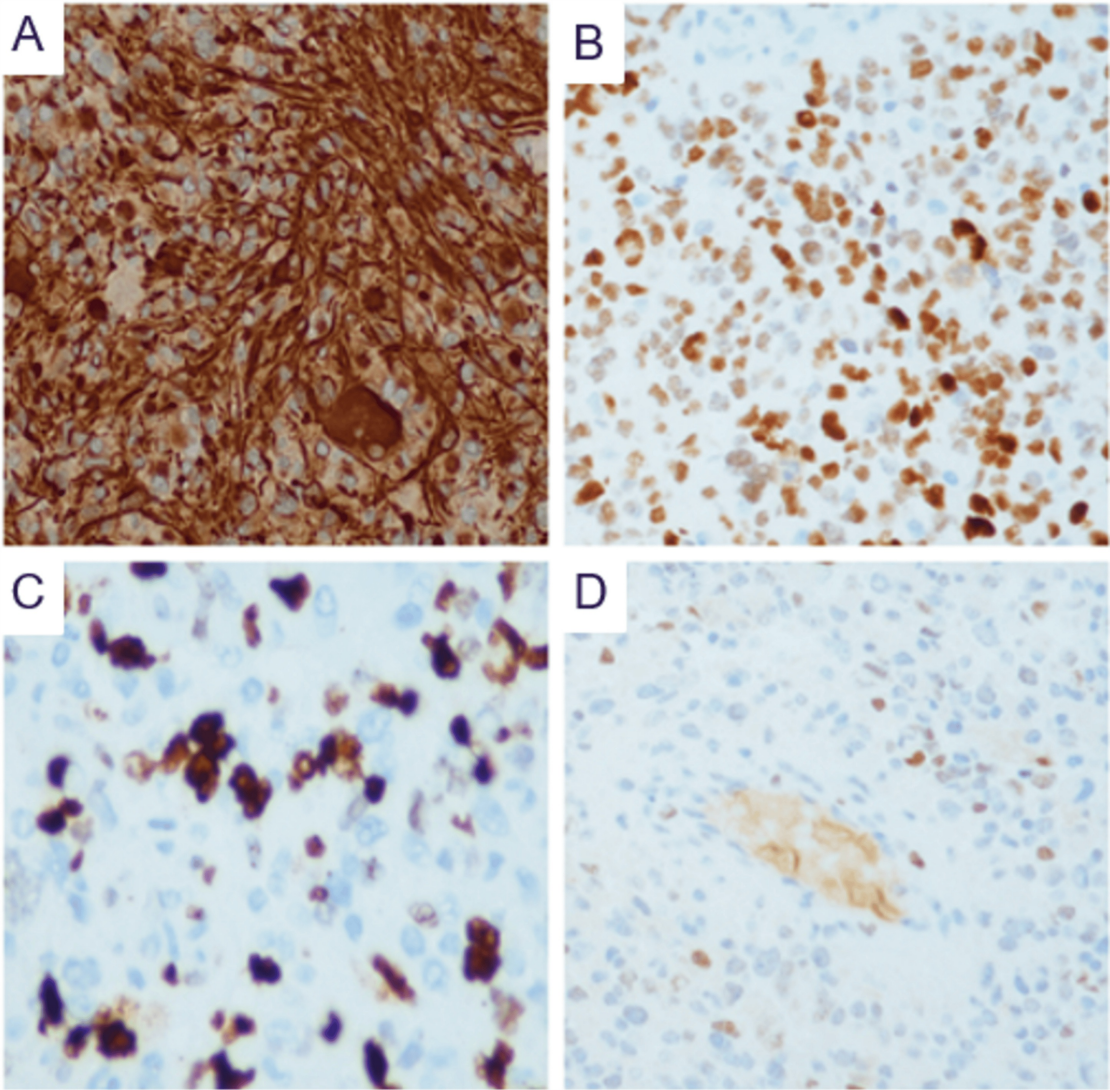
Immunohistochemical profile of the brain tumor (A) GFAP showing strong, diffuse cytoplasmic positivity in tumor cells (×200). (B) p53 showing strong nuclear positivity in approximately 80% of tumor cells (×200). (C) Ki-67 with a high proliferative index (30%-40% nuclear positivity) (×400). (D) MSH6 showing partial loss of nuclear expression in both tumor and adjacent normal cells (×200).

IHC was performed on formalin-fixed paraffin-embedded tumor sections (4 µm thick) using standard protocols. The following antibodies were applied: GFAP (polyclonal), Olig2 (EP112), p53 (DO-7), Ki-67 (MIB-1), IDH1 R132H (MRQ-67), ATRX (polyclonal rabbit), BRAF V600E (VE1), and H3K27M (LYZ27). For MMR proteins, antibodies included PMS2 (EP51), MSH2 (FE11), MSH6 (EP49), and MLH1 (ES05). IHC supported the diagnosis, demonstrating positivity for GFAP, Olig2, and p53 (80%), along with a high proliferative index (Ki-67: 30%-40%). IDH1 R132H, ATRX, BRAF V600E, and H3K27M were negative. MMR protein expression showed partial loss of MSH6 in both tumor and adjacent normal cells, with retained MLH1, PMS2, and MSH2 (Figure 4).

In view of these findings, molecular testing was performed on peripheral blood using the CentoCancer® panel, which employs next-generation sequencing (NGS) targeting cancer predisposition genes. Germline analysis identified a VUS in the MSH6 gene (c.3680T>G, p.Ile1227Arg), with no additional actionable pathogenic variants detected (Table 1).

**Table 1:** Genetic analysis results from CentoCancer® panel This table summarizes the germline MSH6 variant (c.3680T>G; p.Ile1227Arg) identified in the patient, showing variant coordinates, amino acid change, zygosity, in silico parameters, allele frequency data, and classification as a variant of uncertain significance (Class 3). Source: [9-15].

Type and classification	Allele frequencies	In silico parameters	Zygosity	SNP identifier	Amino acid change	Variant coordinates	Gene
Missense uncertain significance (Class 3)	gnomAD, –; ESP, –; 1000G, –; CentoMD®, –	PolyPhen-2, probably damaging; Align-GVDG, N/A; SIFT, –; MutationTaster, disease causing; Conservation_nt, high; Conservation_aa, –	Homozygous	N/A	p.(Ile1227Arg)	NM_000179.2:c.3680T>G	MSH6

PCR-based MSI testing showed a stable result.

During the same hospitalization, the patient developed new respiratory symptoms, and imaging revealed a mediastinal mass. Biopsy of the lesion confirmed TLL. The final diagnosis included diffuse pediatric-type high-grade glioma, H3-wildtype and IDH-wildtype (WHO Grade 4), and TLL.

**Figure 5:**
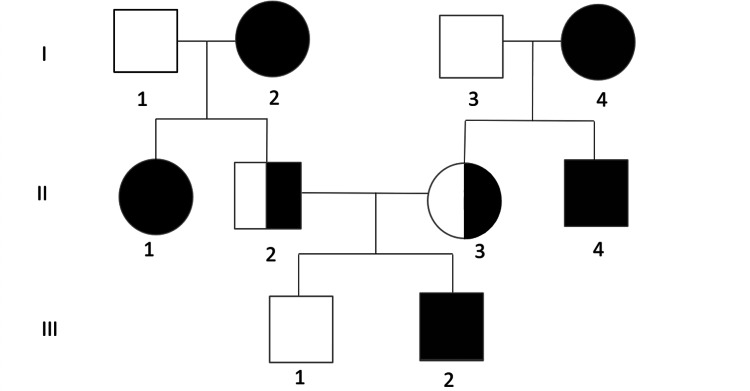
Pedigree of the family (I-1) Paternal grandfather, unaffected. (I-2) Paternal grandmother, brain tumor (deceased). (I-3) Maternal grandfather, unaffected. (I-4) Maternal grandmother, colorectal cancer. (II-1) Paternal aunt, endometrial cancer. (II-2) Father, heterozygous carrier, asymptomatic. (II-3) Mother, heterozygous carrier, asymptomatic. (II-4) Maternal uncle, brain tumor. (III-1) Healthy sibling. (III-2) Proband, CMMRD with high-grade glioma and T-lymphoblastic lymphoma. Image Credit: Authors' original creation.

The patient was born to first-cousin parents and has one healthy sibling. Family history was notable for multiple malignancies on both sides: the paternal grandmother had a malignant brain tumor and is deceased, the maternal grandmother was diagnosed with colorectal cancer, the paternal aunt had endometrial cancer, and the paternal uncle had brain tumor (Figure 5).

This clustering of malignancies within the family, combined with consanguinity and early-onset tumors, raised a strong suspicion of a hereditary cancer predisposition syndrome. Segregation analysis, performed on the patient and his parents, demonstrated that both parents were heterozygous carriers of the same MSH6 variant, while the patient was homozygous. These findings support autosomal recessive inheritance, consistent with a diagnosis of CMMRD (Table 2).

**Table 2:** Segregation analysis of the patient and his parents The patient was homozygous for the variant, while both parents were heterozygous carriers.

Phenotype	Variant characteristics	Genotype	Individual
Symptomatic	Homozygous	MSH6 variant (c.3680T>G, p.Ile1227Arg)	Patient
Asymptomatic carrier	Heterozygous	MSH6 variant (c.3680T>G, p.Ile1227Arg)	Mother
Asymptomatic carrier	Heterozygous	MSH6 variant (c.3680T>G, p.Ile1227Arg)	Father

Although the identified MSH6 variant is classified as a VUS, the combination of clinical features, family history, biallelic involvement, and partial loss of MSH6 expression by IHC supports the diagnosis of CMMRD.

Following the diagnosis, the patient subsequently received focal brain radiotherapy (50.9 Gy in 30 fractions) and induction chemotherapy for TLL according to the COG AALL1231 protocol, which included vincristine, cyclophosphamide, cytarabine, and bortezomib, with omission of 6-MP and weekly intrathecal therapy. By the end of induction, radiologic evaluation demonstrated an 87% reduction in the size of the mediastinal mass, while the glioma showed radiologic progression. Consolidation chemotherapy was administered concurrently with focal cranial irradiation. Immune checkpoint inhibitors (ICIs) were not initiated, as the multidisciplinary tumor board prioritized achieving remission of the aggressive lymphoma before considering ICI therapy for the glioma. The overall prognosis remains poor due to the aggressive nature of both tumors and the underlying cancer predisposition syndrome (CMMRD).

## Discussion

CMMRD-associated gliomas can present with multifocal intracranial lesions, which are not the result of metastatic spread but arise from a constitutional defect in DNA MMR affecting all somatic cells. This germline defect produces a hypermutator phenotype and a “field cancerization” effect within the CNS, predisposing multiple regions to develop independent primary tumors over time. The European Consortium Care for CMMRD (C4CMMRD) series documented multiple synchronous or metachronous brain tumors in pediatric patients, sometimes with associated developmental venous anomalies and cortical malformations [16].

From a histopathological perspective, a notably high proportion (approximately two-thirds) of CMMRD-associated gliomas display giant, multinucleated tumor cells on hematoxylin-eosin staining, reflecting the severe genomic instability and abnormal mitotic control driven by the underlying MMR deficiency [17]. These distinct microscopic features, while highly suggestive, require integration with established clinical and genetic diagnostic frameworks.

Within this framework, diagnostic criteria for CMMRD have been established by consensus groups including the International Replication Repair Deficiency Consortium (IRRDC) and the European Consortium C4CMMRD. According to these criteria, a pediatric or young adult patient should be evaluated for CMMRD if they accumulate three or more points based on a weighted scoring system. Points are assigned for the presence of hallmark malignancies (e.g., high-grade glioma, T-cell lymphoma), additional features including café-au-lait macules, pilomatricomas, brain malformations, a second childhood tumor, Lynch spectrum cancer in a relative, or parental consanguinity. Achieving the threshold indicates the need for molecular and ancillary testing to confirm CMMRD [1,18]. In contrast, Lynch syndrome results from monoallelic (heterozygous) pathogenic variants in the same genes and typically presents in adulthood with a narrower tumor spectrum [1].

Building on this context, the present case highlights the diagnostic and clinical significance of a homozygous MSH6 variant (c.3680T>G; p.Ile1227Arg), currently classified as a VUS in ClinVar (Variation ID: 1832434) [19]. To date, this variant has not been previously reported in the homozygous state. The clinical presentation in this patient, dual malignancy, consanguinity, early age of onset, café-au-lait macules, and immunohistochemical loss of MSH6, closely aligns with the phenotype of CMMRD, supporting the variant’s pathogenicity under the American College of Medical Genetics and Genomics (ACMG) criteria.

Specifically, this case fulfills the following ACMG criteria: PM3 (strong), confirmed homozygosity in a recessive condition; PP1 (supporting), co-segregation with disease with both parents identified as heterozygous carriers; PM2 (moderate), extremely low allele frequency in population databases (two alleles in gnomAD) [12]; and PP4 (supporting), a phenotype highly specific to CMMRD, including early-onset glioma, hematologic malignancy, consanguinity, and café-au-lait macules [1]. 

However, even with such supportive genetic evidence, diagnostic pitfalls remain. This case illustrates one such challenge: the tumor exhibited microsatellite stability (MSS) despite immunohistochemical loss of MSH6 expression. This mirrors known limitations of PCR-based MSI testing in MSH6-associated brain tumors, where the low mitotic index may result in undetectable microsatellite errors by conventional assays [1]. Therefore, reliance on MSI testing alone in CNS tumors may delay accurate diagnosis.

Recognizing CMMRD at an early stage is crucial for guiding treatment, implementing surveillance strategies, and providing genetic counseling. While surveillance in sporadic cancers typically starts in adulthood, guidelines such as those by GENTURIS/C4CMMRD recommend biannual brain MRI, annual gastrointestinal screening, and whole-body imaging beginning in early childhood, especially crucial in consanguineous populations [20].

Therapeutically, the implications are significant. Traditional chemotherapy (e.g., alkylating agents) may have limited efficacy or increased toxicity in hypermutated, MMR-deficient tumors. In contrast, ICIs such as anti-PD-1 therapies have shown clinical benefit in hypermutated gliomas and hematologic malignancies in CMMRD patients [21].

Ultimately, this case contributes novel clinical and genetic data supporting reclassification of the MSH6 variant toward likely pathogenic status. It underscores the importance of multidisciplinary evaluation, including neuropathology, IHC, radiology, and molecular genetics, in children presenting with syndromic cancer patterns, while also emphasizing the need for heightened awareness and clinical suspicion in resource-limited settings where consanguinity is common.

## Conclusions

This case report documents CMMRD associated with a homozygous MSH6 variant (c.3680T>G; p.Ile1227Arg), which has not been previously reported in the homozygous state. The combination of clinical, pathological, and genetic findings provides strong evidence supporting its potential reclassification as likely pathogenic. However, definitive classification requires evaluation by an accredited diagnostic laboratory.

Beyond its molecular relevance, the case highlights the broader importance of early recognition of inherited cancer syndromes in pediatric patients, particularly in communities with high consanguinity. Early identification of CMMRD plays a critical role in guiding clinical management, enabling appropriate surveillance protocols, and providing essential information for family counseling and risk assessment. Continued efforts to report and characterize such variants are crucial for improving outcomes in affected populations.

## References

[1] Carrato C, Sanz C, Muñoz-Mármol AM (2021). The challenge of diagnosing constitutional mismatch repair deficiency syndrome in brain malignancies from young individuals. Int J Mol Sci.

[2] (2025). MedlinePlus. Constitutional mismatch repair deficiency syndrome. https://medlineplus.gov/genetics/condition/constitutional-mismatch-repair-deficiency-syndrome/.

[3] Özyörük D, Cabı EÜ, Taçyıldız N (2021). Cancer and constitutional mismatch repair deficiency syndrome due to homozygous MSH 6 mutation in children with café au lait spots and review of literature. Turk J Pediatr.

[4] Sait SF, Walsh MF, Karajannis MA (2021). Genetic syndromes predisposing to pediatric brain tumors. Neurooncol Pract.

[5] Tan S, Wu X, Wang A, Ying L (2021). Diagnostic challenges in a CMMRD patient with a novel mutation in the PMS2 gene: a case report. BMC Med Genomics.

[6] Brodeur GM, Nichols KE, Plon SE, Schiffman JD, Malkin D (2017). Pediatric cancer predisposition and surveillance: an overview, and a tribute to Alfred G. Knudson Jr. Clin Cancer Res.

[7] Kroeze E, Weijers DD, Hagleitner MM (2022). High prevalence of constitutional mismatch repair deficiency in a pediatric t-cell lymphoblastic lymphoma cohort. Hemasphere.

[8] Teachey DT, Pui CH (2019). Comparative features and outcomes between paediatric T-cell and B-cell acute lymphoblastic leukaemia. Lancet Oncol.

[9] Adzhubei IA, Schmidt S, Peshkin L (2010). A method and server for predicting damaging missense mutations. Nat Methods.

[10] Ng PC, Henikoff S (2003). SIFT: predicting amino acid changes that affect protein function. Nucleic Acids Res.

[11] Schwarz JM, Cooper DN, Schuelke M, Seelow D (2014). MutationTaster2: mutation prediction for the deep-sequencing age. Nat Methods.

[12] Karczewski KJ, Francioli LC, Tiao G (2020). The mutational constraint spectrum quantified from variation in 141,456 humans. Nature.

[13] The 1000 Genomes Project Consortium (2015). A global reference for human genetic variation. Nature.

[14] Tennessen JA, Bigham AW, O'Connor TD (2012). Evolution and functional impact of rare coding variation from deep sequencing of human exomes. Science.

[15] (2025). CentoMD® Variant Database. https://www.centogene.com/.

[16] Guerrini-Rousseau L, Varlet P, Colas C (2019). Constitutional mismatch repair deficiency-associated brain tumors: report from the European C4CMMRD consortium. Neurooncol Adv.

[17] Guerrini-Rousseau L, Merlevede J, Denizeau P (2024). Glioma oncogenesis in the constitutional mismatch repair deficiency (CMMRD) syndrome. Neurooncol Adv.

[18] Wimmer K, Kratz CP, Vasen HF (2014). Diagnostic criteria for constitutional mismatch repair deficiency syndrome: suggestions of the European consortium 'care for CMMRD' (C4CMMRD). J Med Genet.

[19] (2025). NIH. NM_000179.3(MSH6):c.3680T>G (p.Ile1227Arg) and hereditary nonpolyposis colorectal neoplasms. https://www.ncbi.nlm.nih.gov/clinvar/RCV001908788.4/.

[20] Colas C, Guerrini-Rousseau L, Suerink M (2024). ERN GENTURIS guidelines on constitutional mismatch repair deficiency diagnosis, genetic counselling, surveillance, quality of life, and clinical management. Eur J Hum Genet.

[21] Le DT, Durham JN, Smith KN (2017). Mismatch repair deficiency predicts response of solid tumors to PD-1 blockade. Science.

